# A Cortical Thickness Mapping Method for the Coxal Bone Using Morphing

**DOI:** 10.3389/fbioe.2018.00149

**Published:** 2018-10-18

**Authors:** J. Sebastian Giudice, David Poulard, Bingbing Nie, Taotao Wu, Matthew B. Panzer

**Affiliations:** Department of Mechanical and Aerospace Engineering, Center for Applied Biomechanics, University of Virginia, Charlottesville, VA, United States

**Keywords:** pelvis, finite element modeling, Kriging, GHBMC, human body modeling, cortical bone

## Abstract

As human body finite element models become more integrated with the design of safety countermeasures and regulations, novel models need to be developed that reflect the variation in the population's anthropometry. However, these new models may be missing information which will need to be translated from existing models. During the development of a 5th percentile female occupant model (F05), cortical thickness information of the coxal bone was unavailable due to resolution limits in the computed tomography (CT) scans. In this study, a method for transferring cortical thickness information from a source to a target model with entirely different geometry and architecture is presented. The source and target models were the Global Human Body Models Consortium (GHBMC) 50th percentile male (M50) and F05 coxal bones, respectively. To project the coxal bone cortical thickness from the M50 to the F05, the M50 model was first morphed using a Kriging method with 132 optimized control points to the F05 anthropometry. This technique was found to be accurate with a mean nodal discrepancy of 1.27 mm between the F05 and morphed M50 (mM50) coxal bones. Cortical thickness at each F05 node was determined by taking the average cortical thickness of every mM50 node, non-linearly weighted by its distance to the F05 nodes. The non-linear weighting coefficient, β, had a large effect on the accuracy and smoothness of the projected cortical bone thickness. The optimal projection had β = 4 and was defined when the tradeoff between projection accuracy and smoothness was equal. Finally, a quasi-static pelvis compression was simulated to examine to effect of β. As β, increased from 0 to 4, the failure force decreased by ~100 N, whereas the failure displacement increased by 0.9 mm. Results from quasi-static compression tests of the F05 pelvis were comparable to experimental results. This method could be applied to other anatomical regions where cortical thickness variation is important, such as the femur and ribs and is not limited to GHBMC-family models. Furthermore, this process will aid the development of subject-specific finite element models where accurate cortical bone thickness measurements cannot be obtained.

## Introduction

Pelvic fracture is a significant cause of disability and mortality (Gokcen et al., 1994; Inaba et al., 2004). Finite element analysis (FEA) is a popular tool for assessing the human response to impact and has been widely used to predict the structural response and injury prediction of the pelvis during high rate blunt trauma (Song et al., 2005; Li et al., 2007). On a broader scale, FEA is widely used in the automotive injury for assessing human body models (HBM) in injurious loading conditions, such as pedestrian and automotive impacts (Nie et al., 2017, 2018; Wu et al., 2017; Chen et al., 2018). These models have a large role as they have a profound impact on the development of new countermeasures and government safety regulations.

The ability to predict pelvic fracture using FEA is largely contingent on an accurate representation of the cortical layer of the coxal bone, which has been shown to vary regionally throughout its structure (Besnault et al., 1998; Anderson et al., 2008; Kim et al., 2009; Salo et al., 2012; Ma et al., 2015). Obtaining accurate cortical thickness measurements from clinical CT images, however, remains problematic due to limitations in resolution and a lack of well-defined boundaries between the cortical and underlying trabecular bone (Prevrhal et al., 1999). Anderson et al. demonstrated that by increasing the radiation dosage beyond clinically safe levels, the coxal bone cortical thickness measurements of a cadaveric specimen could be made with less than 10% error (Anderson et al., 2005). This method was limited to cadaveric pelves as radiation doses are strictly regulated in the clinical setting and is only practical for subject specific models where cadaveric CT images are used for both thickness calculations and to define the FE model geometry.

Applying cadaveric cortical thickness data to a clinically-generated FE model is challenging given that cortical thickness is spatially dependent and different pelves vary in anatomy. Some models, such as the Global Human Body Model Consortium (GHBMC) owned 50th percentile male occupant model (M50), contain cortical thickness information in the coxal bone (Anderson et al., 2005). In an effort to increase the spectrum of HBM, the GHBMC have created additional HBM with different anthropometry based on volunteer anatomical scans, but limitations of the scan limited cortical thickness measurements to select long bones, and excluded the pelvis and ribs (Davis et al., 2014).

Thus, a method that accounts for anatomical variation is required to transfer the cortical thickness information of a one HBM to another. Recently, Kim et al. (2014), proposed a method to project cortical thickness information from a source (Anderson et al., 2005) to a target (GHBMC M50, Gayzik et al., 2011) FE model. The proposed method involved using a shape-preserving parameterization algorithm to map the two models into a common space and then transferring the nodal information from each source node to the analogous target node using point-wise interpolation (Kim et al., 2014). This technique was effective because both source and target pelves were male, and the models had similar nodal architecture and mesh topology, making the transfer of information relatively straight-forward. However, if the target model were female, anatomical differences would be more prominent due to strong sexual dimorphism of the pelvis (Coleman, 1969; Wang et al., 2004). Additional complications would exist if the target model already had an existing mesh with different mesh topology.

This was the case with the development of the GHBMC-owned 5th percentile female occupant (F05) model. The objective of this study was to develop a technique to transfer the cortical thickness from a source FE model (GHBMC M50) to a target model (GHBMC F05) while preserving the target model mesh topology and nodal architecture. This was achieved by utilizing a Kriging technique (Trochu, 1993) to morph the source to the geometry of the target to facilitate a direct transfer of cortical thickness.

## Materials and methods

### Model descriptions

The source and target coxal bones were extracted from the GHBMC 50th percentile male (M50-O v4.3) and 5th percentile female (F05-O v2.0) occupant full body FE models (Gayzik et al., 2011; Davis et al., 2014). In the M50 model, coxal cortical bone was represented with a single layer of penta- and hexahedral solid elements. To represent the variation in cortical thickness, nodes on the inner and outer surfaces were mapped to the endo- and periosteal surfaces of the CT scans. Therefore, the cortical thickness of each element was represented by the distance between its inner and outer surfaces. The initial F05 model represented the cortical layer of the coxal bone using constant thickness triangular shell elements.

### Source model thickness calculation

**Figure 1:**
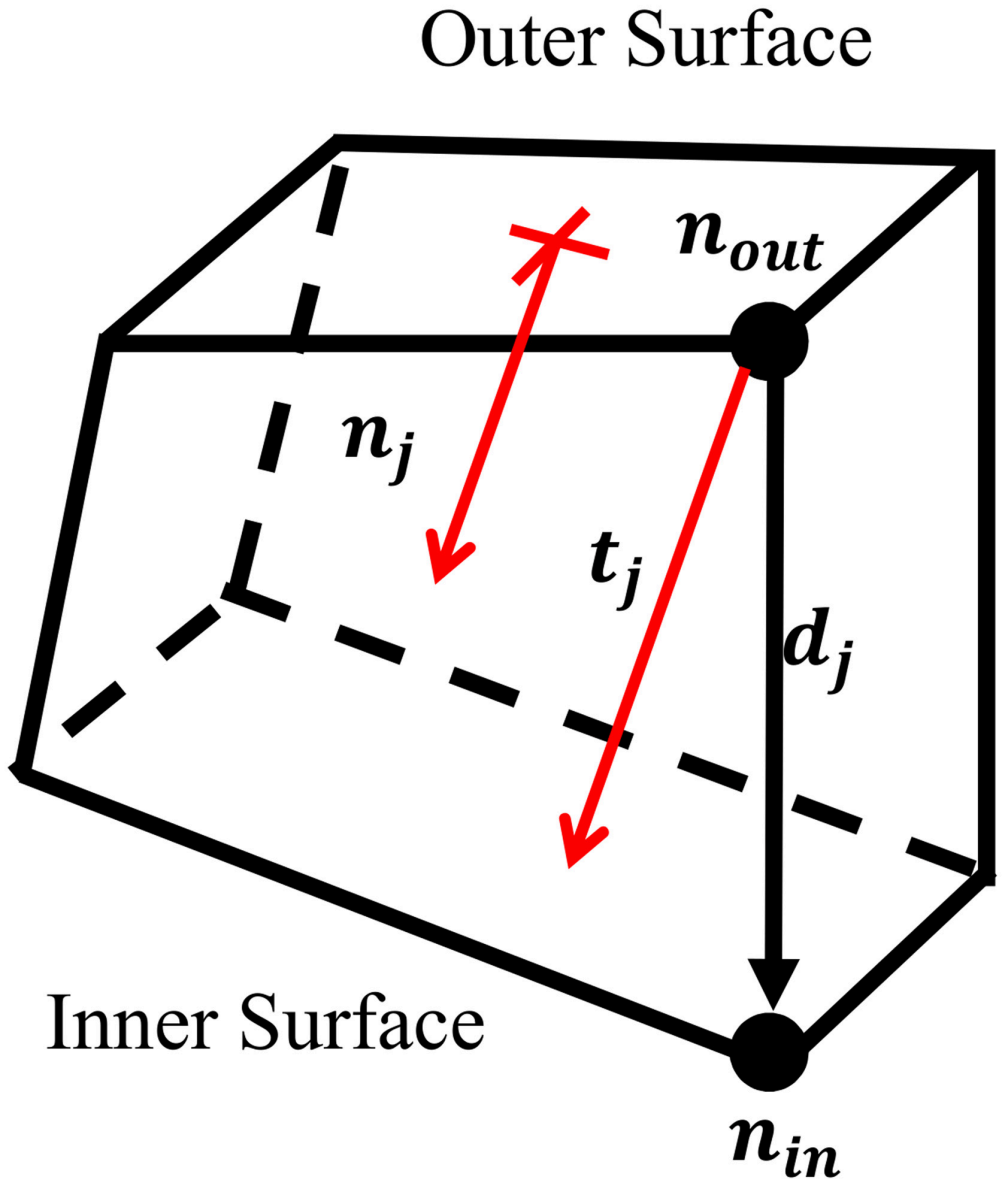
Adjustment of thickness vector.

To project the thickness of the M50 model, the thickness at each node on the outer surface of the solid coxal cortical bone was calculated. For each node on the outer surface, *n*_*out*_, the corresponding node on the inner surface, *n*_*in*_, was found. Using the coordinates of each inner and outer node pair, a direction vector, ***d***_*j*_, was defined, but was not always in the direction of the thickness of the cortical bone. To account for this, a unit vector perpendicular to the outer surface, ***n***_*j*_, was created. By projecting ***d***_*j*_ upon ***n***_*j*_, the fraction of the direction vector in the direction of the normal vector, α, was found. This correction factor was used to scale the direction vector, resulting in a perpendicular thickness vector for each outer node, ***t***_*j*_. Nodal thickness (thickness measured at each outer surface node) was taken as the magnitude of ***t***_*i*_ (Figure 1).

### Morphing

Morphing was performed using a Kriging technique which has been widely utilized to personalize FE models to subject-specific geometries (Besnault et al., 1998; Vezin and Berthet, 2009; Jolivet et al., 2015; Park et al., 2017; Chen et al., 2018). Kriging morphs an initial mesh to a target mesh given two sets of control points. A total of 142 anatomical landmarks were hand-selected as morphing control points in the M50 and F05 coxal bones. Hand-selecting these points allowed for accurate representation of key anatomical landmarks of the pelvis, including the anterior superior iliac spine (ASIS), anterior inferior iliac spine (AIIS), greater sciatic notch, pubic tubercle, ischial tuberosity, pubic rami, and acetabulum. The point selection was then optimized to provide the most accurately morphed model. This optimization was performed by removing each control point one-by-one and recomputing the morphing algorithm. The root-mean-square error (RMSE) was calculated between the F05 and corresponding morphed M50 (mM50) model control points for each morph and was used to determine which control points introduced error to the morph. These control points were discarded and a total of 132 control points were used in the final, optimized morph (Figure 4A).

For each control point, *r*, with coordinates, ***x***_*r*_ = (*x*_*r*_, *y*_*r*_, *z*_*r*_), selected from meshes *i* (i.e., F05) and *j* (i.e., M50), Kriging assesses a linear drift function plus a function of distances between the control point *r* and the other points *s* (fluctuation):

$$\begin{array}{l} x_{r}^{j}=\underset{drift}{\underset{︸}{a_{0}^{x}+a_{1}^{x}x_{r}^{i}+a_{2}^{x}y_{r}^{i}+a_{3}^{x}z_{r}^{i}}}+\underset{fluctuation}{\underset{︸}{\overset{n}{\underset{s=1}{\sum }}b_{s}^{x}K\left(\left||x_{r}^{i}-x_{s}^{i}|\right|\right)}} \end{array}$$

where $a_{i}^{x}$ and $b_{s}^{x}$ are constants, and *n* is the number of points, *s*. The generalized covariance function, *K*, was a logarithmic function of the distance $\left||x_{r}^{i}-x_{s}^{i}|\right|$ and is widely used in the fields of medical imaging, human body modeling (Trochu, 1993; Holden, 2008), and soft-tissue artifact compensation (Dumas and Cheze, 2009).

$$\begin{array}{l} {K\left(\left||x_{r}^{i}-x_{s}^{i}|\right|\right)=\left(\left||x_{r}^{i}-x_{s}^{i}|\right|\right)}^{2}\log\left(\left||x_{r}^{i}-x_{s}^{i}|\right|\right) \end{array}$$

Other forms of the generalized covariance function (linear, cubic, Gaussian, multi-quadratic, and inverse multi-quadratic) were assessed prior to this study and the logarithmic form was found to result in the best morph with respect to element quality, algorithm efficiency, and morph accuracy.

### Nodal thickness projection

Once the M50 pelvis was morphed to the F05 geometry, the morphed M50 nodal thicknesses were projected to the nodes of the F05 cortical bone shell elements using a non-linear weighted average function. The distance, *q*_*ij*_, was calculated for every *ith* node on the F05 cortical shell, with coordinates $x_{i}^{F}$, to every *jth* node on the outer surface of the mM50 cortical layer, with coordinates $x_{j}^{M}$. The distance measure was used as a weighting factor to calculate the thickness at the given F05 node based on the thickness in the mM50 cortical layer, where the influence of the source nodes diminished as the distance to the target nodes increased. Control of the influence of each node in the non-linear weighting scheme is based on the coefficient β, which effectively filters the thickness mapping (increasing β yielded a noisier thickness projection). As β increased, the influence of faraway source nodes decreased, and as β approached infinity, the weighted average converged upon a nearest neighbor solution. For β = 0, the average thickness of the source model was projected uniformly to every node of the target geometry.

$$\begin{array}{l} q_{ij}=|{\text{s}}_{i}|=|x_{i}^{F}-x_{j}^{M}| \end{array}$$

$$\begin{array}{l} t_{i}^{F}\left(\beta \right)=\frac{\sum_{j=1}^{N}t_{j}{\left(\frac{1}{q_{ij}}\right)}^{\beta }\;}{\sum_{j=1}^{N}{\left(\frac{1}{q_{ij}}\right)}^{\beta }\;} \end{array}$$

Where $t_{i}^{F}$is the thickness of the *ith* F05 cortical shell node, β is the non-linear weighting coefficient (β ≥ 0), *N* is the number of mM50 outer surface cortical nodes, *t*_*j*_ is the thickness of the *jth* mM50 node, ***s***_*i*_ is the vector from the *ith* F05 cortical shell node to the *jth* mM50 node, and *q*_*ij*_ is the magnitude of ***s***_*i*_. To investigate the effect of β, numerous projections were calculated with β = 0–4, 10, 25, and 50. These values of β were chosen to investigate the sensitivity of β on the ensuing cortical thickness projection. Between 0 and 4 this parameter was found to nonlinearly influence the projection accuracy and smoothness, and the β = 50 projection converged to the nearest-neighbor solution. The methods used to investigate the influence of β on projection accuracy and smoothness are discussed in the following section.

Cortical bone thickness was represented in the F05 model using the ^*^ELEMENT_SHELL_THICKNESS keyword in LS-Dyna (LSTC, Livermore, CA, USA) which allows users to define thickness for each 2D shell element with nodal resolution (Hallquist, 2006, 2007). In FEA, shell thickness is built into the fundamental equations of the stiffness calculations based on plate bending theory. This prevented cusps in the model surface that would have otherwise occurred if each original shell element was extruded in its normal direction to create solid elements.

### Projection accuracy and smoothness

Because the meshes between the target and the source are different, there is not a direct node-to-node mapping of cortical thickness value. Using a nearest-neighbor approach to mapping the values would yield the most consistent thickness distribution between the two meshes, but may result in large thickness gradients in the mesh that could lead to artificial stress concentrations in the numerical solution. Thus, for each thickness projection, two metrics were used to evaluate the mapping process: accuracy and smoothness. Accuracy quantified the variation between the cortical thickness values in the target geometry to the cortical thickness values in the source geometry, and smoothness quantified the relative change in cortical thickness value between neighboring nodes in the target geometry. To assess the accuracy of the thickness projection, the thickness distributions for each β were compared to the projection with β = 50. Since this projection most resembled a nearest-neighbor method, it was assumed that it had a perfect accuracy of 1.

$$\begin{array}{l} A\left(\beta \right)=\left(1-\frac{\sum_{i=1}^{P}{\left(t_{i}^{F}\left(50\right)-t_{i}^{F}\left(\beta \right)\right)}^{2}}{\sum_{i=1}^{P}{\left(t_{i}^{F}\left(50\right)-t_{i}^{F}\left(0\right)\right)}^{2}}\right) \end{array}$$

Where *A*(β) is the accuracy of the projection and a function of β, *P* is the number of F05 nodes, and $t_{i}^{F}\left(\beta \right)$ is the thickness of the *ith* node and a function of β.

Model smoothness was analyzed using a three-dimensional multivariate linear regression. To be clear, smoothness is a measure of how noisy the mapped cortical shell thickness values are, and not a measure of the roughness of the F05 pelvis mesh. For each node, a linear regression was generated to predict the thickness values of the connected nodes based on their coordinates. This regression model assumes a first order gradient of cortical shell thickness over the surface of the pelvis.

$$\begin{array}{l} {\hat{t}}_{m}^{i}=C_{1}x_{m}+C_{2}y_{m}+C_{3}z_{m}+C_{4} \end{array}$$

Where ${\hat{t}}_{m}^{i}$, is a *m* × 1 vector of the predicted thicknesses of the *m* nodes connected to the current node, *i, C*_1_*-C*_4_ are regression parameters, and ***x***_*m*_, ***y***_*m*_, and ***z***_*m*_, are *m* × 1 vectors of the *x, y*, and *z* Cartesian coordinates of the *m* attached nodes. The predicted thickness was calculated for each attached node and the regression error between the predicted thickness and projected thickness was determined.

$$\begin{array}{l} E_{i}\left(\beta \right)=|\;{\hat{t}}_{m}^{i}-t_{m}^{i}{|}^{2} \end{array}$$

Where *E*_*i*_(β) is the regression error between the thickness predicted by the first order regression model, ${\hat{t}}_{m}^{i}$ and $t_{m}^{i}$, which is a *m* × 1 vector of the projected thicknesses of the *m* nodes connected to the current node, *i*. For a perfectly smooth projection (β = 0), there is no discrepancy between the predicted and actual thickness and all *E*_*i*_(0) = 0 since the thicknesses are identical. As β increases, the mapped thickness values become noisier, and the mapped thicknesses values deviate from the first order gradient predicted by the linear regression model. This results in the regression error of the *ith* node to increase. This process was repeated for all *i* nodes in the model and the overall smoothness metric was calculated in a similar manner to the accuracy.

$$\begin{array}{l} S\left(\beta \right)=\left(\frac{\sum_{i=1}^{P}{\left(E_{i}\left(50\right)-E_{i}\left(\beta \right)\right)}^{2}}{\sum_{i=1}^{P}{\left(E_{i}\left(50\right)-E_{i}\left(0\right)\right)}^{2}}\right) \end{array}$$

Where *S*(β) is the smoothness of the model and a function of β and ranges from 0 in the constant thickness projection (β = 0) to 1 in the nearest-neighbor projection (β = 50).

### Model evaluation

**Figure 2:**
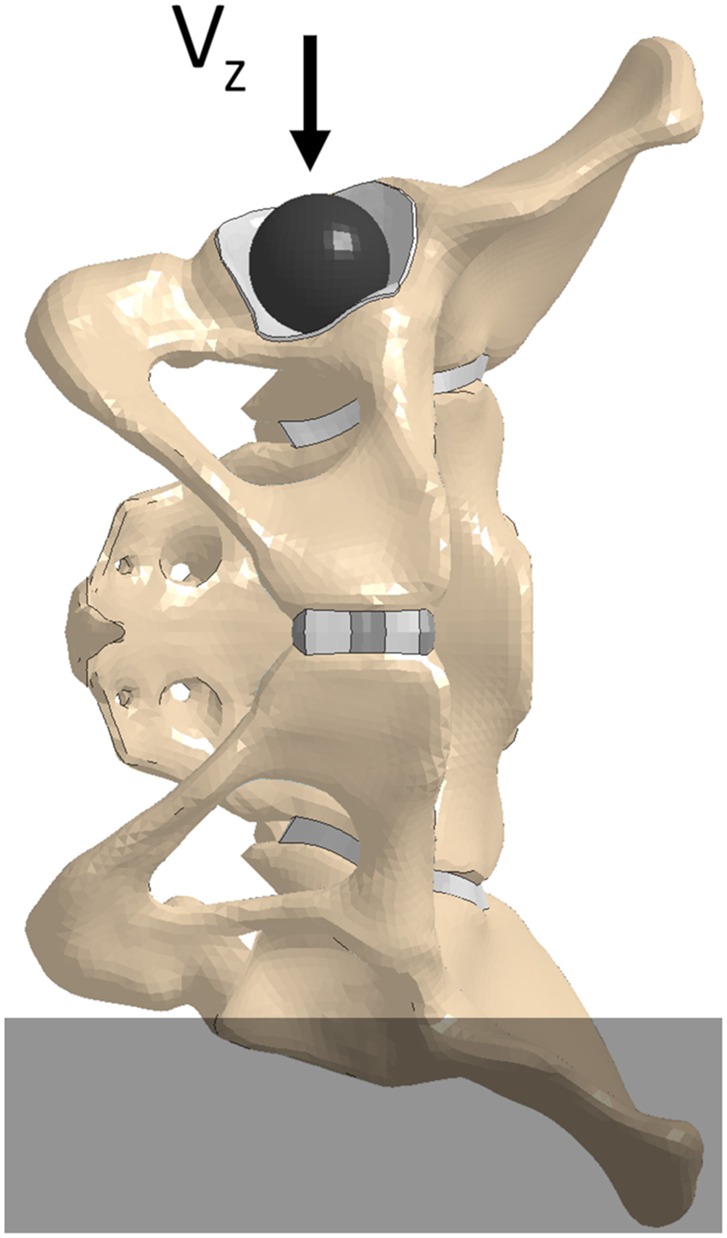
Model set up for quasi-static lateral compression of the pelvis.

A quasi-static lateral pelvis compression (Guillemot et al., 1998) simulation was executed to investigate the effect of β on the pelvic response. The pelvis' structural components were extracted from the GHBMC F05-O full body model and were positioned with the left iliac wing constrained up to the external edge of the left ischial tuberosity. A rigid shell sphere was created to match the geometry of the ipsilateral acetabulum and was placed within it. To prevent point loading, a thin “cartilaginous” pad (1 mm thick) was extruded from the acetabulum shell elements and assigned elastic material properties (Figure 2). To minimize inertial effects, and maintain the quasi-static boundary condition, a loading rate of 5 mm/min was utilized in the experimental test protocol. The material properties of the F05 pelvis were modified to remove strain-rate dependence and a compressive loading rate of 5,000 mm/min was prescribed to the rigid sphere in the—z direction (downwards, Figure 2). The contact force and rigid body displacement of the sphere were defined as outputs. All simulations were performed using LS-Dyna v7.1.1.

## Results

### Source model thickness calculation

**Figure 3:**
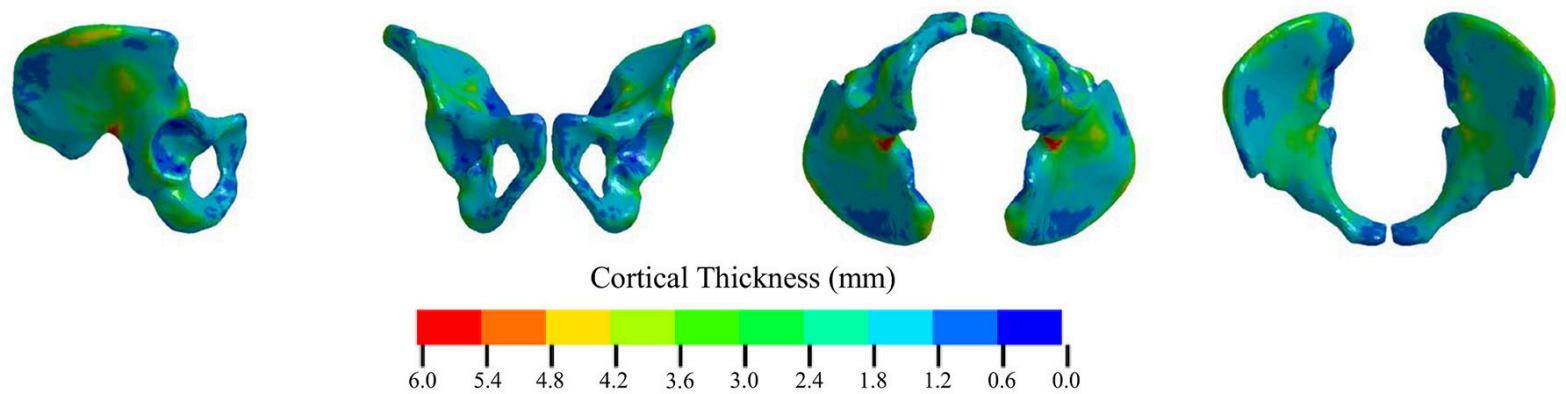
Calculated cortical thickness of the unmorphed GHBMC M50 pelvis.

Figure 3 shows the calculated cortical thickness for the M50 pelvis. The greatest cortical thickness was 8.33 mm and was observed in the greater sciatic notch. The smallest cortical thickness was 0.29 mm and was located on the anterior inferior surface of the acetabulum. The mean calculated cortical thickness was 1.77 ± 0.69 mm.

### Morphing

**Figure 4:**
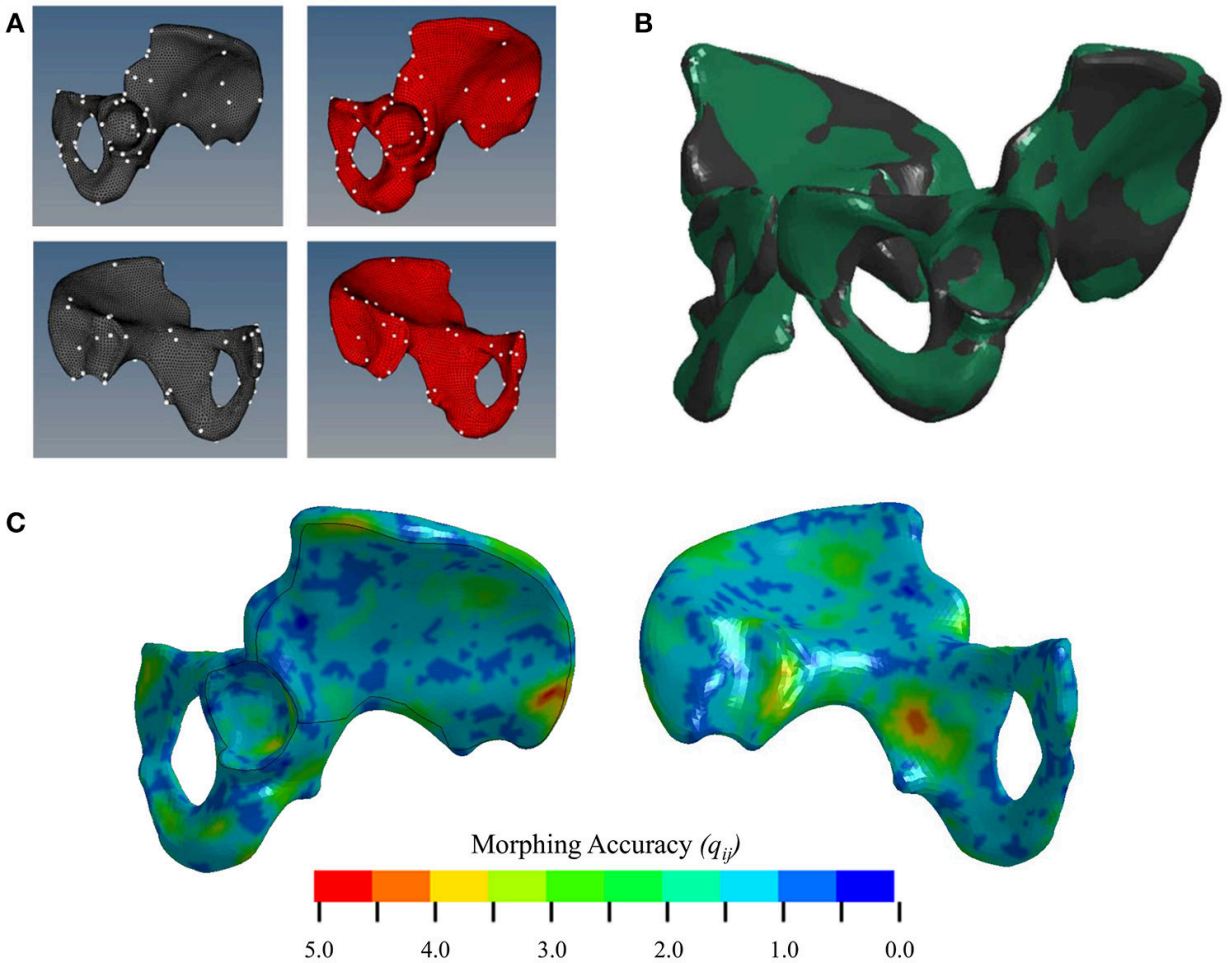
**(A)** Selection of control points for Kriging on the lateral (top) and medial (bottom) surfaces of the coxal bone. **(B)** Kriging results. Black—F05; Green—mM50 (morphed). **(C)** Morphing accuracy measured by the distance (mm) between corresponding F05 and M50 (morphed) nodes.

To facilitate the transfer of cortical bone thickness data from the source to the target model, the source pelvis was morphed using a Kriging technique. For every node on the F05 cortical shell, the average distance to its corresponding M50 node, *q*_*ij*_, before Kriging was 12.4 ± 4.54 mm. Table 1 shows that by morphing the M50 model to F05 anthropometry, *q*_*ij*_ was reduced by an order of magnitude. The initial morph utilized a collection of 142 anatomical landmarks to serve as control points. The optimal morph solution reduced the number of control points to 132 (Figure 4A). While the average *q*_*ij*_ was similar for the initial and optimized morph solutions, the maximum *q*_*ij*_ and root mean squared error (RMSE) were reduced through the optimization process. Figure 4B shows a side-by-side comparison of the mM50 and F05 coxal bones. Figure 4C demonstrates *q*_*ij*_ variation throughout the entire surface of the F05 model. The region of greatest *q*_*ij*_ was located on the posterior superior iliac spine followed by the medial surface of the ischium, superior iliac crest, posterior surface of the acetabulum, and anterior pubis.

**Table 1:** Morphing results for the source model.

**Morph (control points)**	***q_*ij*_* (mm)**	**max(*q_*ij*_*) (mm)**	**RMSE**
Initial (142)	1.32 ± 0.42	8.65	179.0
Optimized (132)	1.27 ± 0.33	4.89	34.9

### Nodal thickness projection

**Figure 5:**
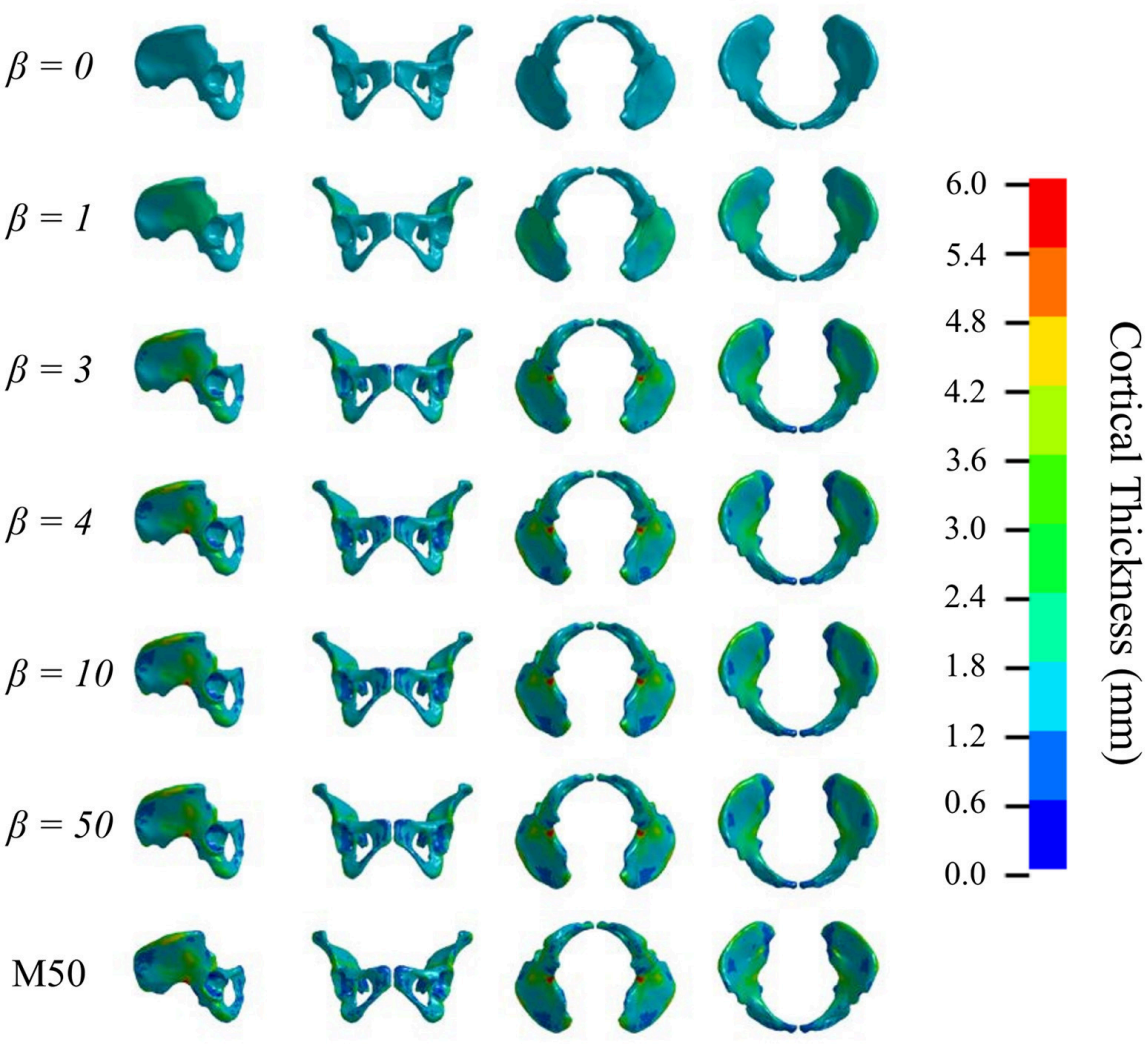
Variation in cortical thickness projection with increasing β. Spatial variation in cortical thickness changes as β increases from 0 to 3. The solution converges at β = 4.

**Figure 6:**
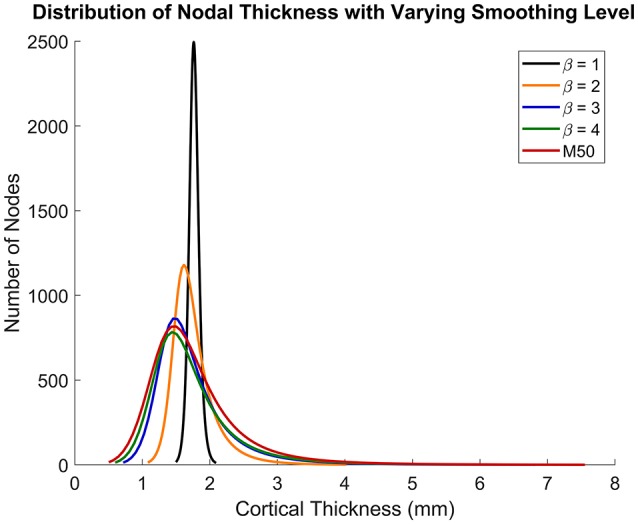
Nodal distribution of cortical thickness as β increases from 1 to 4.

Figures 5, 6 show that the non-linear coefficient, β had an effect on the distribution of the projected thickness map between β = 1–4. Within this range, the largest differences in projected cortical bone thickness were observed along the iliac crest, sciatic notch, AIIS, and the posterior inferior iliac spine (PIIS). β had the smallest effect on the thicknesses of the pubic rami, ischium, and the iliac fossa (Figure 5). To investigate this effect, a kernel smoothing function was utilized to generate distribution curves for thickness projections with β = 1–4, which were then compared to the source model's distribution (Figure 6). As β increased beyond 4, the solution converged upon a single, nearest-neighbor solution. β values of 10, 25, and 50 did not demonstrate noticeable differences in the projected thickness distribution and were omitted from Figures 5, 6.

### Projection accuracy and smoothness

**Figure 7:**
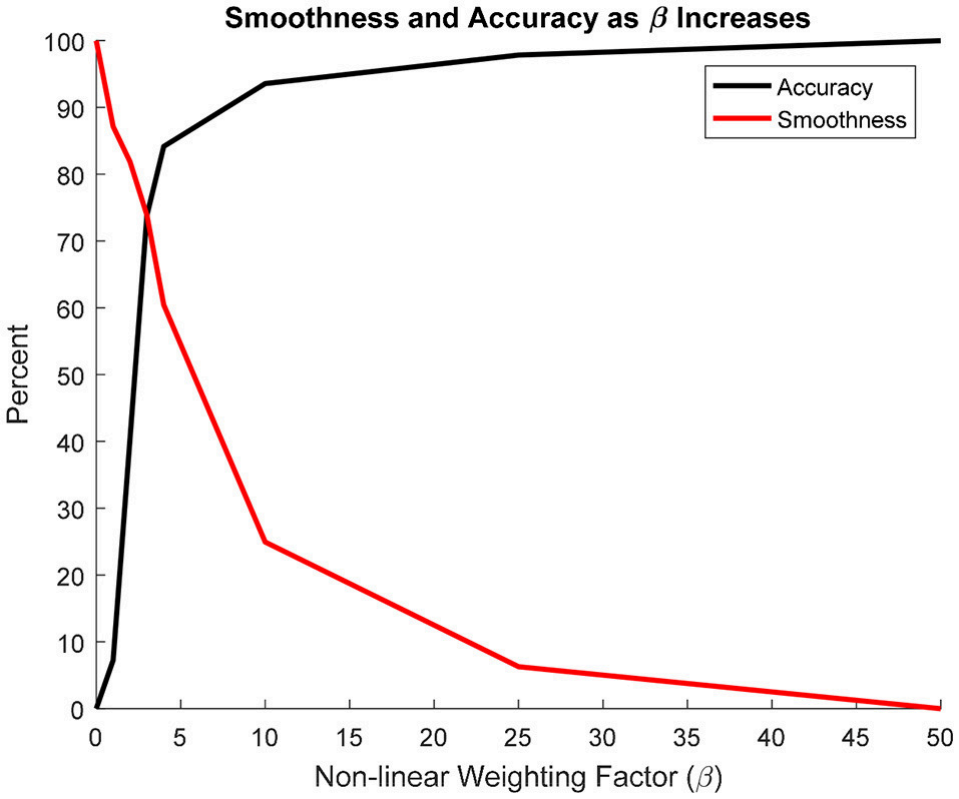
Change in projection accuracy and smoothness as β increased from 0 to 50. Smoothness and accuracy intersect at approximately β = 3.

To quantify the accuracy of each projection, it was assumed that the β = 50 projection was most accurate since it most resembled a nearest-neighbor projection. Figure 7 shows that increasing β led to an exponential increase in accuracy. Conversely, as β increased, smoothness decreased exponentially. The intersection between the accuracy and smoothness curves occurred between β = 3 and 4.

### Model evaluation

**Figure 8:**
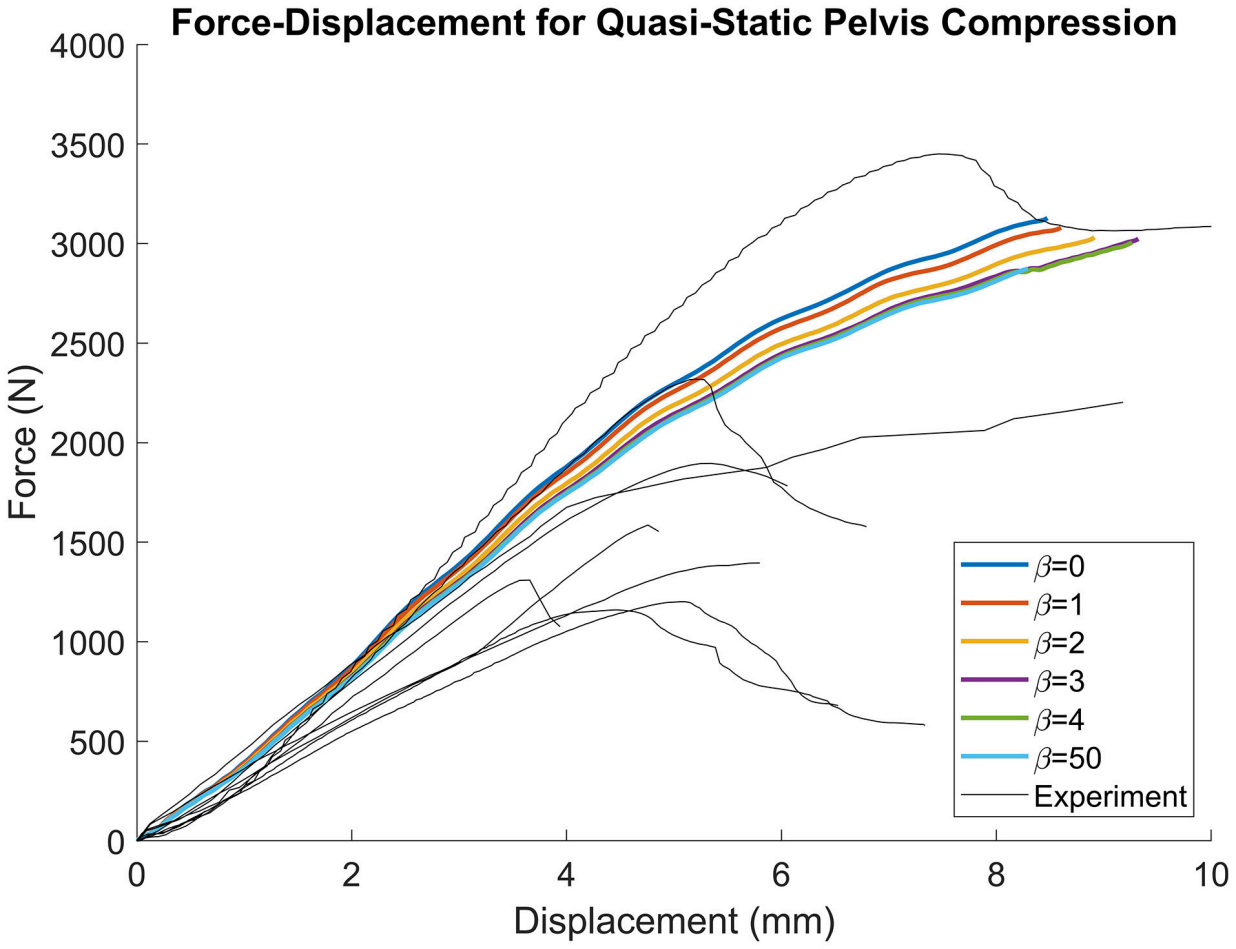
Force-displacement results for quasi-static lateral pelvis compression. Traces converge as β increases, but the failure force decreases with increasing β.

Figure 8 demonstrates that as β increased from 0 (constant thickness) to 4 the force-displacement history converged upon a steady-state solution. Furthermore, as β increased, the failure force decreased from 3,128 (β = 0) to 3,024 N (β = 4) and failure displacement increased from 8.476 (β = 0) to 9.321 mm (β = 4). For the β = 50 simulation, the failure force decreased from 3,062 to 2,873 N and the failure displacement decreased from 9.39 to 8.296 mm. For all simulations, failure was located at the inferior and ischial pubic rami, contralateral to loading. Negligible variation in the number of elements or location of failure was observed. All simulation results were comparable to the experimental results (Figure 8). Experimental failure forces were between 1,100 and 3,400 N and failure displacements between 3.5 and 7.5 mm (Guillemot et al., 1998).

## Discussion

The objective of this study was to develop a method to transfer the cortical bone thickness from a source model to a target model while preserving the target's nodal architecture and topology. While a similar method was developed to transfer information between similar pelvis meshes (Kim et al., 2014), the novelty of the current method was that it was suitable for dissimilar meshes in both anthropometry and topology. The current method was used in the development of a female pelvis model (GHBMC F05) from a male pelvis model (GHBMC M50), which was complicated by the fact that the two models had different nodal architecture and topologies. Specifically, the source model utilized a single layer of hexahedral and pentahedral elements to represent the cortical layer of the coxal bone, whereas the target model used triangular shell elements. This task was accomplished by calculating the cortical thickness of each M50 solid element at the nodal level, employing a Kriging technique to morph the source model to the anatomy of the target, and subsequently using a nonlinear weighting function to project cortical bone thickness.

The ability to project cortical thickness from one model to another is contingent on geometric similarity. A Kriging technique (Trochu, 1993) was utilized to morph the M50 pelvis to the anatomy of the F05 model. Kriging is a common technique used in the computational biomechanics field and was chosen due to its efficiency, accuracy, and ability to preserve element quality (Besnault et al., 1998; Serre et al., 2006; Jolivet et al., 2015). Control points were selected using anatomical landmarks that could be identified in both M50 and F05 models and subsequently optimized to yield the most accurate morph solution. In general, morphing accuracy was best in regions where control points were easily identified, such as the AIIS. Conversely, morphing accuracy was poorest in amorphous regions, such as the posterior iliac crest and medial ischial body, where control points could not be defined (Figure 4C). Therefore, to improve morphing accuracy, a repeatable and robust technique for selecting control points in amorphous regions of the pelvis is necessary. This technique has the potential to be used for other anatomy regions where control points could be easily identified or their extraction could be automated (Poulard et al., 2012).

To our knowledge, this is the first study to use a morphing technique to project non-material model parameters (in this case, cortical bone thickness) to a model with different anthropometry without modifying the target model architecture and topology. The measured cortical thicknesses of the M50 pelvis varied between 0.294 and 8.332 mm with a mean of 1.770 ± 0.692 mm. However, ~95% of the measured M50 cortical thickness values were between 0.294 and 3.176 mm. The mean M50 cortical thickness was in general agreement with the cortical thicknesses calculated in the studies of Ma et al. (0.52–3.36 mm), Anderson et al. (0.44–4.00 mm), Dalstra et al. (0.7–3.2), and Renaudin et al. (0.5–4.00 mm), however, the maximum thickness found in this study was greater than those previously reported. This is a result of the solid element thicknesses in the M50 pelvis and not of the thickness projection technique presented in this study. Female-specific cortical bone thickness values are not reported in the literature.

There were several limitations to this study. Firstly, this method was dependent upon Kriging to facilitate the projection of cortical thickness from the source to the target model, and projection accuracy was ultimately governed by the accuracy of the morph. Accuracy of the morphing method is often dependent on the control points used, and these may be subject to inter-user variability. However, since the projected thickness distribution of the β = 50 projection, which was closest to a nearest-neighbor approach, was nearly identical to the original M50 thickness distribution (Figures 5, 6), the morph accuracy obtained in this study was sufficient. Secondly, experimental data on the cortical bone thickness of a small female coxal bone was not available for validating the applicability of male coxal cortical bone thickness values in a small female model. The mapping method was developed specifically because of the lack of information available. In the absence of cortical thickness measurement, this study relied on the assumption that the cortical bone thickness distribution between two individuals of different sex and stature were the same. The validity of this assumption is unknown in the pelvis given the lack of data. However, since our goal is to develop a realistic surrogate model for mechanical analysis, and not a subject-specific model (which is also limited by unknown material properties), we believe that mapping variable cortical thickness data from one subject to another is an improvement in biofidelity relative to assuming a constant cortical thickness. Finally, the models were only evaluated using a single load case, without accounting for anatomical differences between the F05 pelvis and the experimentally tested specimens. Further simulations are necessary to characterize the model response's sensitivity to β during dynamic loading conditions and in different loading directions.

In conclusion, this study presented a method for transferring the cortical thickness from a source to a target model with entirely different anatomy and nodal topology. Morphing was used to assimilate the anatomies and a weighted average was utilized to project the cortical thickness from the morphed source to the target model. The accuracy and smoothness of the projection was dependent on the value of the non-linear weighting coefficient, β and this tradeoff was found to be even when β was between 3 and 4. Similarly, the source thickness distribution fell between the β = 3 and 4 distributions and the model response in quasi-static lateral pelvis compression converged when β = 4. Other values of β could also be used if an emphasis on either accuracy or smoothness is desired for a particular application. While an application for the coxal bone of the pelvis was presented, this method could easily be applied to other body regions where local variations in cortical thickness are important, such as the femur and ribs. Additionally, this method is not limited to GHBMC-family models and could be applied to any two models regardless of the mesh architecture. Having a common cortical thickness source model can be useful in developing subject specific models of live volunteers since accurate cortical thickness measurements cannot be consistently made from the CT scans of living subjects.

## Author contributions

All authors contributed to the conception, design, and interpretation of results presented in this study and are accountable for all aspects of this work. JSG developed the cortical bone mapping technique and performed all finite element model simulations, data analysis, and the writing of the manuscript. DP contributed to the morphing of the M50 model.

### Conflict of interest statement

The authors declare that the research was conducted in the absence of any commercial or financial relationships that could be construed as a potential conflict of interest.
